# The Individual and Combined Effects of the Cyanotoxins, Anatoxin-a and Microcystin-LR, on the Growth, Toxin Production, and Nitrogen Fixation of Prokaryotic and Eukaryotic Algae

**DOI:** 10.3390/toxins11010043

**Published:** 2019-01-15

**Authors:** Mathias Ahii Chia, Benjamin J. Kramer, Jennifer G. Jankowiak, Maria do Carmo Bittencourt-Oliveira, Christopher J. Gobler

**Affiliations:** 1 Department of Botany, Ahmadu Bello University, Zaria 810001, Nigeria; 2 School of Marine and Atmospheric Sciences, Stony Brook University, Southampton, NY 11968, USA; benjamin.j.kramer@stonybrook.edu (B.J.K.); jennifer.jankowiak@stonybrook.edu (J.G.J.); 3 Department of Biological Sciences, Luiz de Queiroz College of Agriculture, University of São Paulo, Av. Pádua Dias, 11, São Dimas, Piracicaba, SP 13418-900, Brazil; mbitt@usp.br

**Keywords:** cyanobacteria, allelopathy, chlorophytes, bioactive secondary metabolites

## Abstract

Globally, eutrophication and warming of aquatic ecosystems has increased the frequency and intensity of cyanobacterial blooms and their associated toxins, with the simultaneous detection of multiple cyanotoxins often occurring. Despite the co-occurrence of cyanotoxins such as microcystins and anatoxin-a (ATX) in water bodies, their effects on phytoplankton communities are poorly understood. The individual and combined effects of microcystin-LR (MC-LR) and ATX on the cyanobacteria *Microcystis* spp., and *Anabaena variabilis* (a.k.a. *Trichormus variabilis*), and the chlorophyte, *Selenastrum capricornutum* were investigated in the present study. Cell density, chlorophyll-a content, and the maximum quantum efficiency of photosystem II (Fv/Fm) of *Microcystis* cells were generally lowered after exposure to ATX or MC-LR, while the combined treatment with MC-LR and ATX synergistically reduced the chlorophyll-a concentration of *Microcystis* strain LE-3. Intracellular levels of microcystin in *Microcystis* LE-3 significantly increased following exposure to MC-LR + ATX. The maximum quantum efficiency of photosystem II of *Anabaena* strain UTEX B377 declined during exposure to the cyanotoxins. Nitrogen fixation by *Anabaena* UTEX B377 was significantly inhibited by exposure to ATX, but was unaffected by MC-LR. In contrast, the combination of both cyanotoxins (MC-LR + ATX) caused a synergistic increase in the growth of *S. capricornutum*. While the toxins caused an increase in the activity of enzymes that scavenge reactive oxygen species in cyanobacteria, enzyme activity was unchanged or decreased in *S. capricornutum*. Collectively this study demonstrates that MC-LR and ATX can selectively promote and inhibit the growth and performance of green algae and cyanobacteria, respectively, and that the combined effect of these cyanotoxins was often more intense than their individual effects on some strains. This suggests that the release of multiple cyanotoxins in aquatic ecosystems, following the collapse of blooms, may influence the succession of plankton communities.

## 1. Introduction

Aquatic ecosystems are characterized by different communities of plants and animals that interact with each other and the abiotic environment. Among the photosynthetic organisms, phytoplankton play a key role in maintaining ecological balance as the base of the aquatic food chain [1,2]. Increasing nutrient concentrations can alter plankton communities and promote the formation of algal and cyanobacterial blooms [3,4,5]. Cyanobacterial blooms are a worldwide environmental problem due the production of cyanotoxins, which include hepatotoxins, neurotoxins, cytotoxins, and dermatoxins which inhibit protein synthesis and phosphatase activities, irreversibly block acetylcholinesterase, promote tumor formation, and cause skin problems, respectively [6,7,8]. Current and future changes in global climatic conditions coupled with increasing eutrophication in aquatic ecosystems are already increasing the frequency and intensity of blooms of noxious cyanobacterial genera including *Microcystis*, *Raphidiopsis*, *Anabaena*, *Nodularia*, *Aphanizomenon*, and *Lyngbya* [5]. Consequently, higher concentrations of cyanotoxins are being detected in aquatic ecosystems, a scenario that may have broad effects on the ecology of plants and animals in different environments [9].

Recently, there has been debate and conflicting results regarding the ability of some cyanotoxins to act as allelochemicals [10,11,12,13,14,15]. First order conclusions indicate that the effects of cyanotoxins on phytoplankton and aquatic macrophytes are dependent on the type of cyanotoxin, the concentration and duration of exposure to the cyanotoxin, physicochemical condition of the aquatic environment or media, and the target strain and/or species [11,16,17,18,19]. To date, the hepatotoxins microcystins (MCs) have been the most studied cyanotoxins, given that they are the most commonly encountered cyanotoxins in aquatic ecosystems worldwide [20]. Microcystins are monocyclic heptapeptides synthesized via nonribosomal peptide synthases [21] and are potent inhibitors of protein phosphatases 1 and 2A in plants and animals [22]. They are most commonly made by species in the genera of *Microcystis*, *Planktothrix*, and *Anabaena* and are primarily retained within cells during cyanobacterial blooms, but are released during cell lysis, and in some cases, during interactions with other plants and animals [23,24]. Anatoxin-a (ATX) is another cyanotoxin that is a neuromuscular blocking agent, and at high levels, causes fatal asphyxia in animals after injection or ingestion [25], induces oxidative stress, and inhibits the growth of some aquatic macrophytes and phytoplankton [11,17,18,19,26]. Anatoxins are produced primarily by species belonging to *Anabaena*, *Aphanizomenon*, *Cylindrospermum*, *Planktothrix*, *Microcystis*, *Oscillatoria*, *Microcoleus*, and *Phormidium* [27]. These characteristics suggest that MCs and ATX have multiple physiological and ecological roles that are yet to be comprehensively understood.

As cyanobacteria blooms intensify globally, water bodies containing more than one cyanotoxin will become more commonplace [28,29,30]. For example, producers of ATX and MCs such as *Anabaena*, and *Microcystis* co-occur in aquatic ecosystems under varying physical and chemical conditions [3,29] and both cyanotoxins have been simultaneously detected in many water bodies [28,29,30,31,32]. Despite these findings, only a few studies have considered the interactive effects of cyanotoxins on phytoplankton species [15], limiting our understanding of their ecological roles. Thus, it is unknown whether the combination of MCs and ATX result in antagonistic, additive, or synergistic effects on phytoplankton communities and other aquatic organisms. A better understanding of the effects of cyanotoxin mixtures on prokaryotic and eukaryotic algae can only be achieved by investigating the individual as well as combined effects of cyanotoxins.

The objective of the present study, therefore, was to assess the individual and combined effect of ATX and microcystin-LR (MC-LR) on multiple prokaryotic and eukaryotic phytoplankton species. While microcystin has multiple congeners, congener MC-LR is one of the most frequently encountered and toxic [27,33]. Cultures of Cyanobacteria (*Microcystis* and *Anabaena*), and Chlorophyta (*Selenastrum capricornutum*) were exposed to the two toxins individually and in combination, and the growth, photosynthetic characteristics, toxin content, nitrogen fixation rates, and enzyme activities within cultures were quantified. One of the *Microcrystis* strains, *Microcystis aeruginosa* LE-3, produces microcystins and the *Anabaena* strain, *Anabaena variabilis* (a.k.a. *Trichormus variabilis*) UTEX B377, fixes nitrogen. The concurrent quantification of multiple physiological characteristics of cultures allowed experimental outcomes to be mechanistically contextualized.

## 2. Results

**Figure 1:**
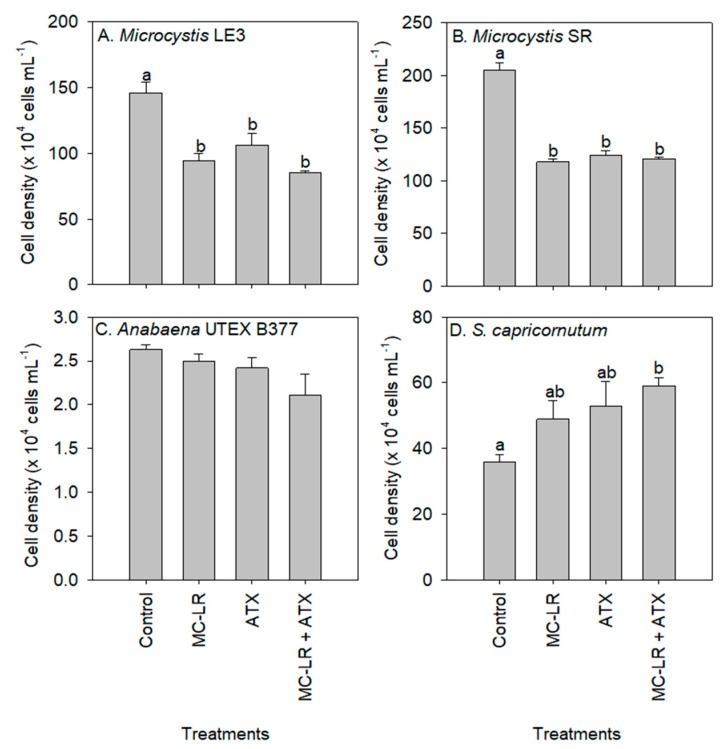
Cell density of (**A**) *Microcystis* LE-3, (**B**) *Microcystis* SR (**C**) *Anabaena* UTEX B377, and (**D**) *Selenastrum capricornutum* exposed to 25 µg L^−1^ microcystin-LR (MC-LR), 25 µg L^−1^ anatoxin-a (ATX) and 25 µg L^−1^ MC-LR + 25 µg L^−1^ ATX (MC-LR + ATX) for four days. Means with different alphabets are significantly different at *p* < 0.05.

Compared to the control (1.50 × 10^6^ cells mL^−1^), there was a decline in the cell density of *Microcystis* LE-3 during exposure to MC-LR (0.94 × 10^6^ cells mL^−1^), ATX (1.06 × 10^6^ cells mL^−1^), and MC-LR+ATX (0.85 × 10^6^ cells mL^−1^) for four days (Figure 1). The lowest cell density of *Microcystis* SR was found in cultures exposed to MC-LR (1.18 × 10^6^ cells mL^−1^), followed by MC-LR + ATX (1.21 × 10^6^ cells mL^−1^) and ATX (1.24 × 10^6^ cells mL^−1^). The inhibition of the growth of *Microcystis* LE-3 and *Microcystis* SR by MC-LR (*p* = 0.01) and ATX (*p* = 0.01) was significant (Figure 1). Furthermore, a significant antagonistic effect of MC-LR and ATX on *Microcystis* cell densities was observed (*p* < 0.05 for *Microcystis* LE-3 and *p* < 0.001 for *Microcystis* SR; Figure 1) because the combination of the toxins did not lead to lower cell densities as would have been expected. While the exposure of the chlorophyte, *S. capricornutum*, to MC-LR and ATX led to higher cell densities than the control, the cultures exposed to ATX and MC-LR + ATX had the most significant increase in cell density (*p* < 0.05; Figure 1). There was a synergistic effect of ATX and MC-LR on *S. capricornutum* as the cultures exposed to the combined treatment (MC-LR + ATX) had higher cell densities than those exposed to the individual treatments (*p* < 0.05; Figure 1).

**Figure 2:**
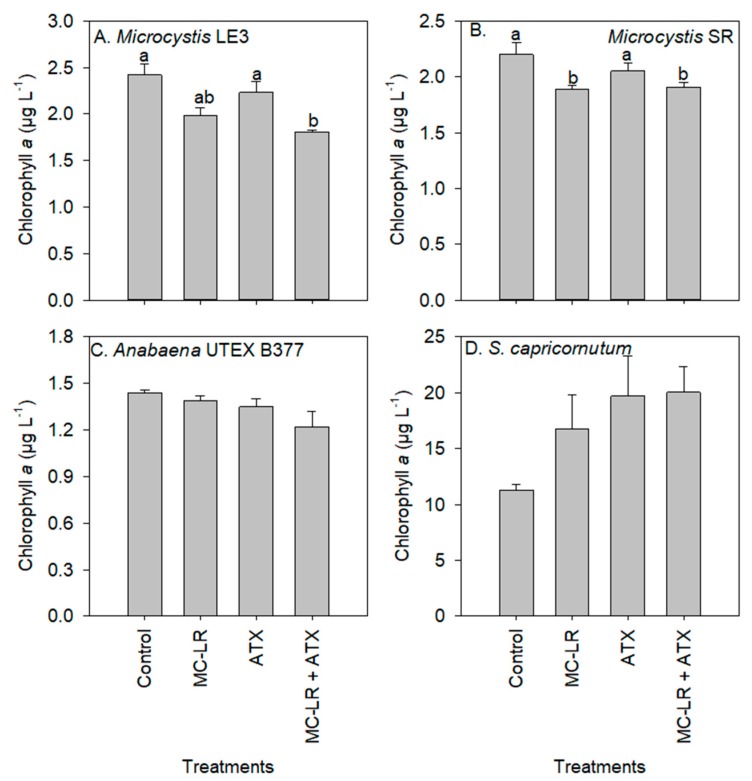
Chlorophyll-a concentration of (**A**) *Microcystis* LE-3, (**B**) *Microcystis* SR, (**C**) *Anabaena* UTEX B377, and (**D**) *Selenastrum capricornutum* after exposure to 25 µg L^−1^ microcystin-LR (MC-LR), 25 µg L^−1^ anatoxin-a (ATX) and 25 µg L^−1^ MC-LR + 25 µg L^−1^ ATX (MC-LR + ATX) for four days. Means with different alphabets are significantly different at *p* < 0.05.

**Figure 3:**
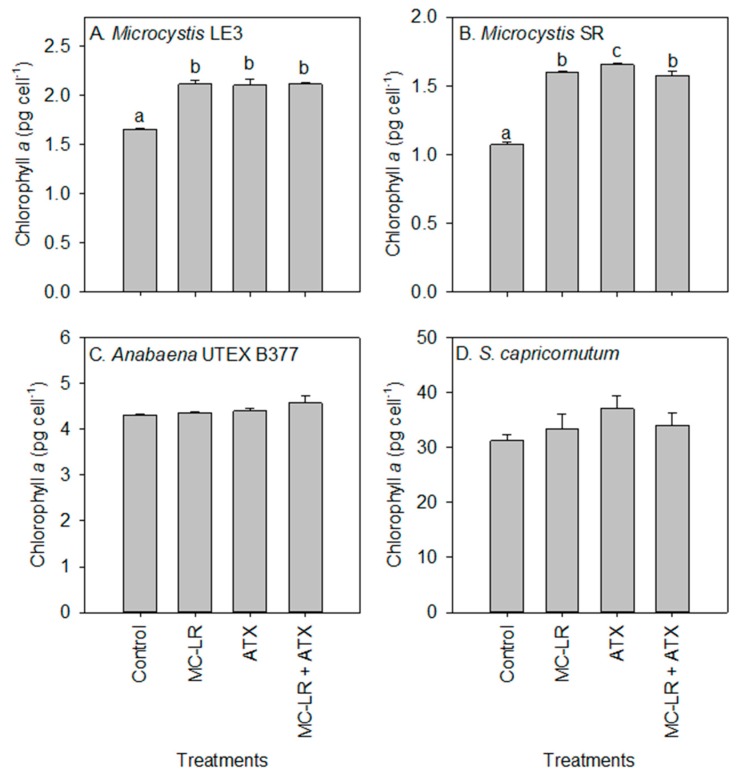
Changes in chlorophyll-a content (pg cell^−1^) of (**A**) *Microcystis* LE-3, (**B**) *Microcystis* SR, (**C**) *Anabaena* UTEX B377, and (**D**) *Selenastrum capricornutum* after exposure to 25 µg L^−1^ microcystin-LR (MC-LR), 25 µg L^−1^ anatoxin-a (ATX) and 25 µg L^−1^ MC-LR + 25 µg L^−1^ ATX (MC-LR + ATX) for four days. Means with different alphabets are significantly different at *p* < 0.05.

Chlorophyll-a concentrations of the *Microcystis* LE-3 (control = 2.42 µg L^−1^) and *Microcystis* SR (control = 2.20 µg L^−1^) cultures were significantly reduced after exposure to MC-LR (1.98 µg L^−1^ and 1.89 µg L^−1^, respectively; *p* < 0.01; Figure 2), while those of cultures exposed to ATX were not significantly altered (2.23 µg L^−1^ and 2.05 µg L^−1^, respectively; *p* > 0.05). There was a general increase in chlorophyll-a content per cell of *Microcystis* LE-3, *Microcystis* SR and *S. capricornutum* after exposure to MC-LR, while that of *Anabaena* UTEX B377 was not. The exposure of *Microcystis* LE-3, *Microcystis* SR and *Anabaena* UTEX B377 to ATX also led to an increase in chlorophyll-a content per cell. Cellular chlorophyll-a content of *Microcystis* cultures exposed to MC-LR, ATX, and MC-LR + ATX was > 40% higher than those of their respective controls (Figure 3). Furthermore, there was a significant antagonistic interaction between MC-LR and ATX on the chlorophyll-a content of the *Microcystis* cultures (*p* < 0.01) whereby the combined treatment did not increase the chlorophyll-a content beyond the individual treatments (Figure 3). The changes in cellular chlorophyll-a content of *Microcystis* cells during exposure to MC-LR (*p* < 0.001) and ATX (*p* < 0.001) were statistically significant, while those recorded in *Anabaena* UTEX B377 and *S. capricornutum* cultures were not (*p* > 0.05; Figure 3).

**Figure 4:**
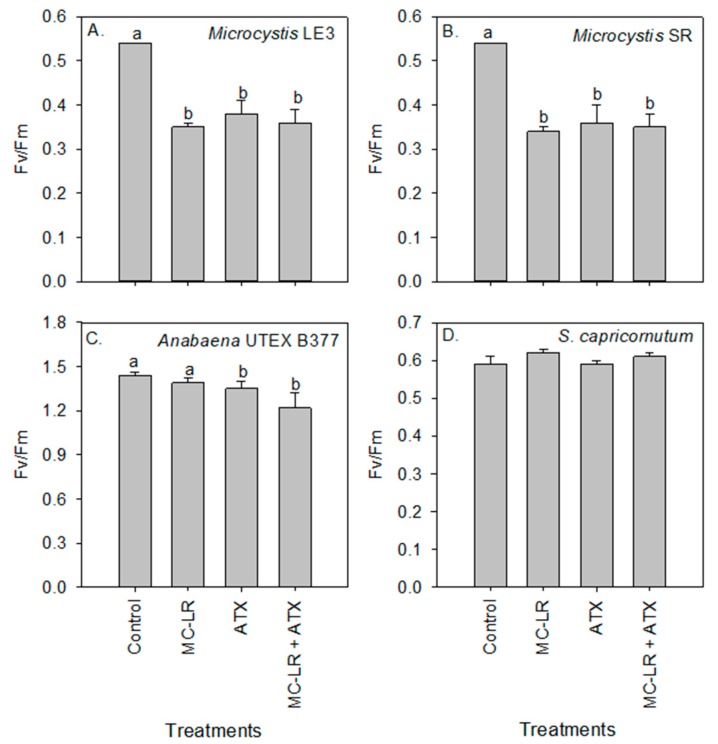
Maximum quantum efficiency of photosystem II (Fv/Fm) values of (**A**) *Microcystis* LE-3, (**B**) *Microcystis* SR, (**C**) *Anabaena* UTEX B377, and (**D**) *Selenastrum capricornutum* on day 4 of exposure to 25 µg L^−1^ microcystin-LR (MC-LR), 25 µg L^−1^ anatoxin-a (ATX), and 25 µg L^−1^ MC-LR + 25 µg L^−1^ ATX (MC-LR + ATX). Means with different alphabets are significantly different at *p* < 0.05.

Maximum quantum efficiency of photosystem II (Fv/Fm) was 33% lower in *Microcystis* LE-3, 30% lower in *Microcystis* SR, and 25% lower in *Anabaena* UTEX B377 during exposure to ATX (*p* = 0.01 for all species; Figure 4). On the other hand, Fv/Fm of *S. capricornutum* was not affected by the treatments (*p* > 0.05). In addition, the reduction of Fv/Fm of *Microcystis* LE-3 and *Microcystis* SR was significant during exposure to MC-LR (37% and 35% reduction, respectively; *p* < 0.001 for both species; Figure 4).

**Figure 5:**
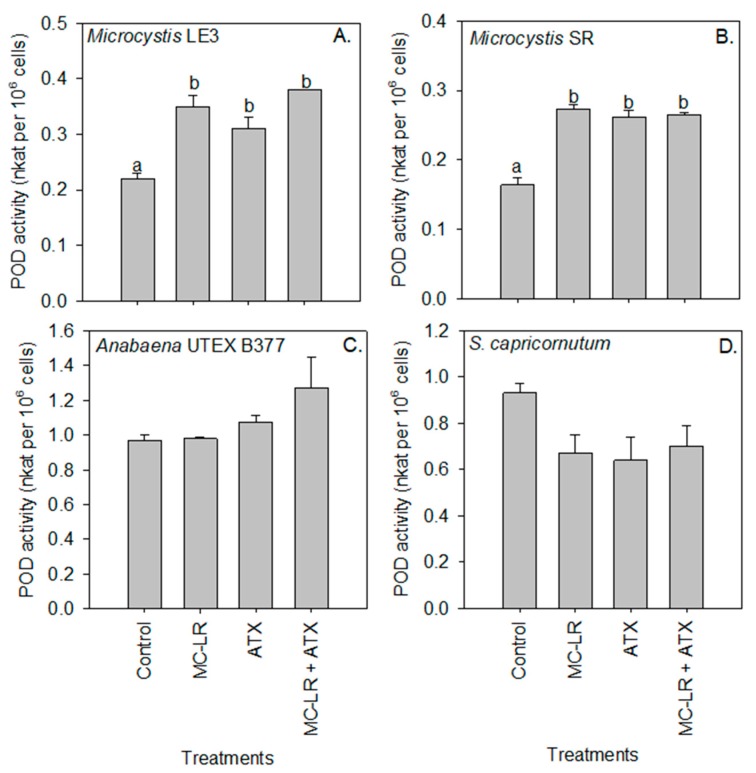
Peroxidase (POD) activity of (**A**) *Microcystis* LE-3, (**B**) *Microcystis* SR, (**C**) *Anabaena* UTEX B377, and (**D**) *Selenastrum capricornutum* exposed to 25 µg L^−1^ microcystin-LR (MC-LR), 25 µg L^−1^ anatoxin-a (ATX), and 25 µg L^−1^ MC-LR + 25 µg L^−1^ ATX (MC-LR + ATX). Means with different alphabets are significantly different at *p* < 0.05.

**Figure 6:**
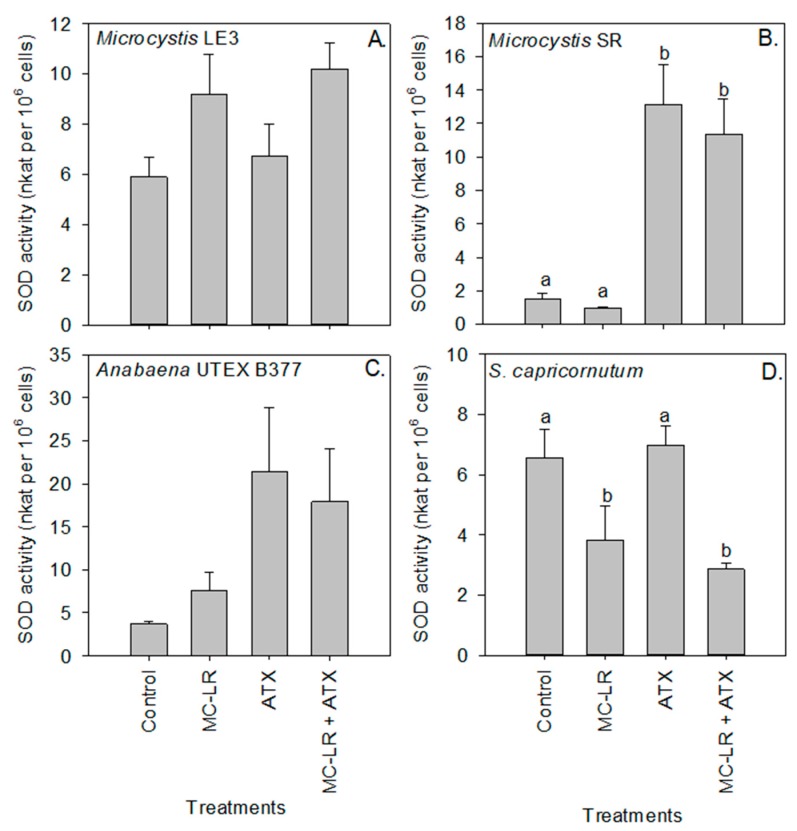
Superoxide dismutase (SOD) activity of (**A**) *Microcystis* LE-3, (**B**) *Microcystis* SR, (**C**) *Anabaena* UTEX B377, and (**D**) *Selenastrum capricornutum* exposed to 25 µg L^−1^ microcystin-LR (MC-LR), 25 µg L^−1^ anatoxin-a (ATX), and 25 µg L^−1^ MC-LR + 25 µg L^−1^ ATX (MC-LR + ATX). Means with different alphabets are significantly different at *p* < 0.05.

Peroxidase activity of *Microcystis* LE-3 (control = 0.22 nKat per 10^6^ cells) and *Microcystis* SR (control = 0.16 nKat per 10^6^ cells) was significantly higher in cultures exposed to ATX (0.31 and 26 nKat per 10^6^ cells, respectively) and MC-LR (35 and 27 nKat per 10^6^ cells, respectively; Figure 5), while that of *Anabaena* UTEX B377 and *S. capricornutum* cultures was not significantly (*p* > 0.05) changed by either of the cyanotoxins. Further, in *Microcystis* SR (*p* < 0.05) an antagonistic interaction was observed between the cyanotoxins on the activity of the peroxidase enzyme. Superoxide dismutase (SOD) activity was significantly (*p* < 0.01) higher in *Microcystis* SR (13.14 nKat per 10^6^ cells) and *Anabaena* UTEX B377 (21.38 nKat per 10^6^ cells) cultures during exposure to ATX compared to the control (3.72 and 1.52 nKat per 10^6^ cells, respectively), while the combination of both cyanotoxins (MC-LR + ATX) had an additive effect on the activity of the enzyme in all the investigated strains (*p* > 0.05; Figure 6).

**Figure 7:**
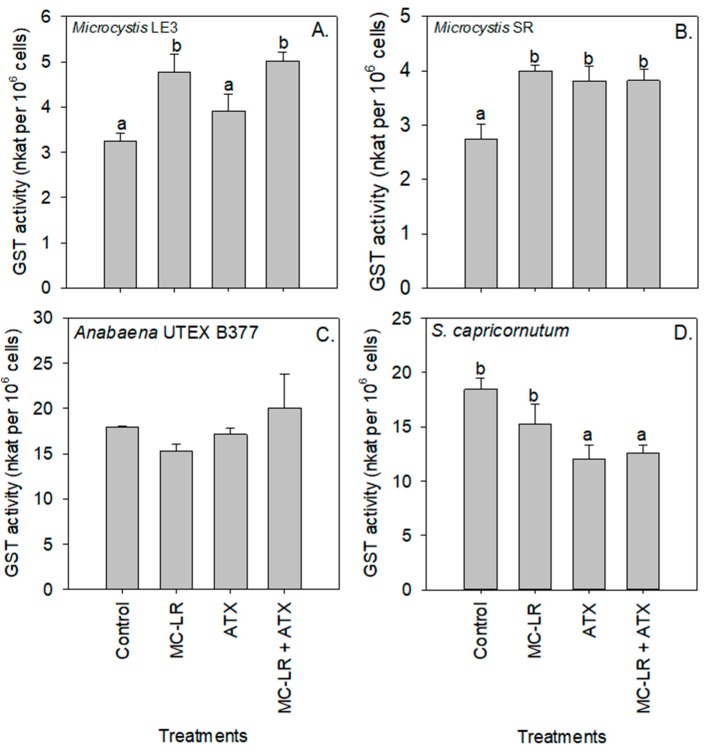
Glutathione S-transferase (GST) activity of (**A**) *Microcystis* LE-3, (**B**) *Microcystis* SR, (**C**) *Anabaena* UTEX B377, and (**D**) *Selenastrum capricornutum* exposed to 25 µg L^−1^ microcystin-LR (MC-LR), 25 µg L^−1^ anatoxin-a (ATX), and 25 µg L^−1^ MC-LR + 25 µg L^−1^ ATX (MC-LR + ATX). Means with different alphabets are significantly different at *p* < 0.05.

The MC-LR treatment caused a significant decrease in the activity of SOD in *S. capricornutum* cells (*p* < 0.01; Figure 6D). Compared to the control, Glutathione S-transferase (GST) activity significantly (*p* < 0.05) increased in *Microcystis* LE-3 and *Microcystis* SR after exposure to both cyanotoxin treatments, with the highest GST activity in *Microcystis* LE-3 (5.02 nKat per 10^6^ cells) recorded in cultures exposed to MC-LR+ATX, and the highest activity in *Microcystis* SR (4.00 nKat per 10^6^ cells) recorded in cultures exposed to MC-LR. GST activity was significantly lower in *S. capricornutum* cultures exposed to ATX (*p* < 0.05; Figure 7). Enzyme activity was not significantly altered by any of the cyanotoxin treatments in the *Anabaena* UTEX B377 cultures (*p* > 0.05 for all treatments, Figure 7).

**Figure 8:**
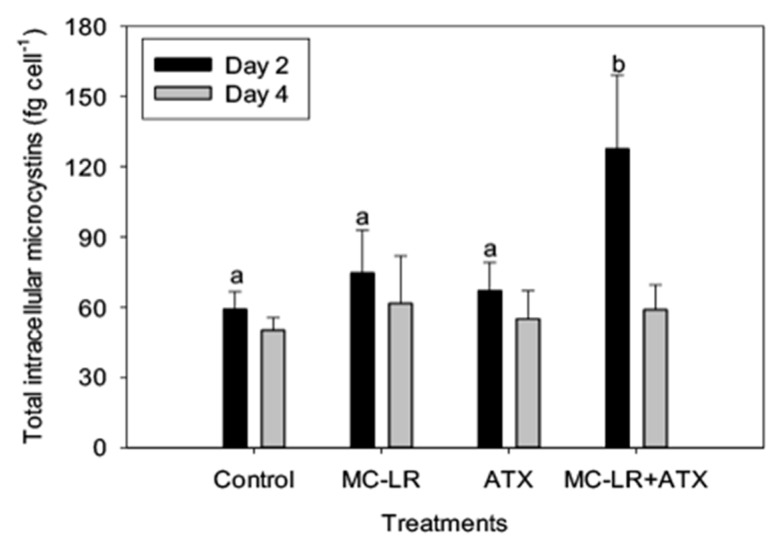
Total intracellular concentrations of microcystins in *Microcystis* LE-3 exposed to 25 µg L^−1^ microcystin-LR (MC-LR), 25 µg L^−1^ anatoxin-a (ATX), and 25 µg L^−1^ MC-LR + 25 µg L^−1^ ATX (MC-LR + ATX). Means with different alphabets are significantly different at *p* < 0.05.

**Figure 9:**
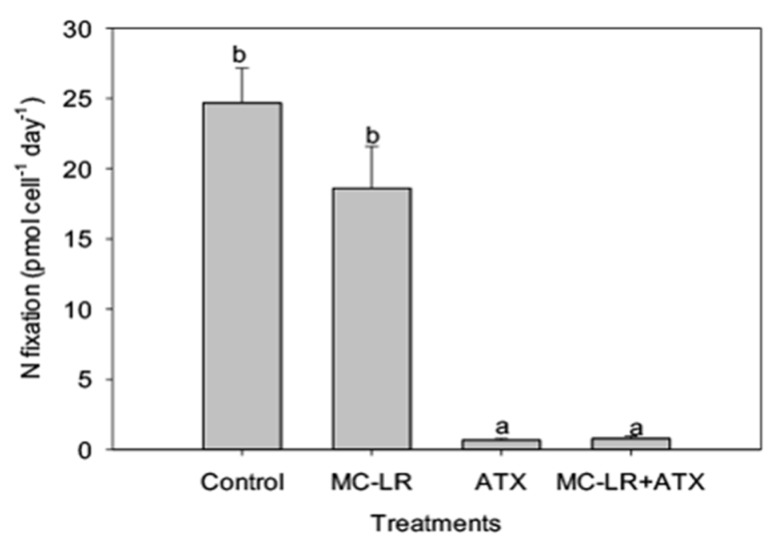
Nitrogen fixation (pmol cell^−1^ day^−1^) of *Anabaena* UTEX B377 exposed to 25 µg L^−1^ microcystin-LR (MC-LR), 25 µg L^−1^ anatoxin-a (ATX) and 25 µg L^−1^ MC-LR + 25 µg L^−1^ ATX (MC-LR + ATX). Means with different alphabets are significantly different at *p* < 0.05.

As *Microcystis* LE-3 is a known producer of MC-LR, total intracellular microcystin concentration of the strain was monitored during the present study. While MC-LR and ATX did not individually alter the MC content of the *Microcystis* LE-3, the combined MC-LR + ATX treatment caused a significant synergistic increase (>50%) in total intracellular MCs content of *Microcystis* LE-3 on day 2 (Figure 8). Among the investigated phytoplankton species, *Anabaena* UTEX B377 represented diazotrophic cyanobacteria. Monitoring changes in nitrogen fixation of this strain provided insights into physiological changes that accompany diazotrophs during exposure to different combinations of cyanotoxins. Nitrogen fixation by *Anabaena* UTEX B377 was the highest in the control treatment, which was not significantly different from the MC-LR treatment (*p* > 0.05; Figure 9). In contrast, exposure to ATX resulted in significantly lower nitrogen fixation by *Anabaena* UTEX B377 compared to the control, with rates declining by more than an order of magnitude (*p* < 0.05; Figure 9).

## 3. Discussion

The frequency, intensity, and extent of toxic cyanobacterial blooms is expanding globally [5] and with this, there is a growing body of literature demonstrating that water bodies with cyanobacterial harmful algal blooms (HABs) can commonly contain multiple cyanotoxins. In the present study, we observed that MC-LR and ATX were capable of altering the growth, pigment production, maximum quantum efficiency of photosystem II, toxin content, antioxidant enzyme activities, and nitrogen fixation rates of non-diazotrophic, diazotrophic, non-toxic, toxic, eukaryotic, and prokaryotic strains of phytoplankton, with the eukaryotic strain often benefiting and prokaryotic strains often being inhibited. These findings have important implications for understanding phytoplankton species succession during and after toxic cyanobacterial blooms.

The growth inhibition of both *Microcystis* strains by the cyanotoxin treatments was consistent with their reduction effect on the maximum quantum efficiency of photosystem II (Figures S1 and S2). These results are similar to those obtained by Phelan and Downing [34], where the exposure of the cyanobacterium *Synechocystis* PCC6803 to exogenous MC-LR led to lower photosystem II activity. Despite an increase in cellular chlorophyll-a content *Microcystis* strains, Fv/Fm values were reduced, thereby limiting the amount of organic substrate produced during photosynthesis [35], which could explain the reduced growth. These results are consistent with field studies that have found that increased chlorophyll-a and phycocyanin content of cyanobacteria does not always lead to higher light use efficiency and maximum quantum efficiency of photosystem II [36]. In field studies of cyanobacteria, the increase in cellular chlorophyll-a content did not compensate for the effects of the cyanotoxin treatments on the maximum quantum efficiency of photosystem II, which is consistent with findings on phytoplankton species exposed to environmental stress [37]. This means that the presence of both cyanotoxins in an aquatic ecosystem is capable of increasing the photosynthetic and physiological stress to which some cyanobacterial species are exposed.

In contrast to the cyanobacteria, *S. capricornutum* experienced a significant increase in chlorophyll-a content per cell, chlorophyll-a concentration per mL, and cell density during exposure to MC-LR and ATX. This outcome is consistent with other recent studies that have documented the resilience of some chlorophytes in the presence of cyanotoxins [14,15,38]. The cyanotoxins seemingly served as growth stimulants to *S. capricornutum* and yielded a synergistic increase in growth and pigment content recorded at the end of the study. The growth stimulation of *S. capricornutum* by ATX and MC-LR is further supported by reports that some green microalgae are able to quickly recover from the effect of cyanotoxins by the activation of efficient defense mechanisms [39]. In addition, prior studies have emphasized the ability of *Selenastrum* spp. to utilize organic compounds for growth [40,41,42]. Hence, *S. capricornutum* may have gained a nutritional benefit from the additional N and C supplied via these compounds. The ability to acquire organic C to supplement photosynthetic C acquisition in dense, potentially light limited cultures would yield significantly more rapid growth than cultures reliant wholly on photosynthesis [43,44,45]. 

While reactive oxygen species are part of the natural metabolism of phytoplankton environmental stress and/or the presence of toxic substances can stimulate excessive production of reactive oxygen species (ROS) in cells [46,47,48]. As a response to increased intracellular ROS levels, phytoplankton can produce antioxidant enzymes such as Peroxidase (POD), SOD, and GST to sequester ROS and therefore mitigate cellular damage caused by these radicals [11,16,49]. In the present study, the activities of all three enzymes were increased in all the cyanobacterial strains following exposure to cyanotoxins, indicating that they were reacting to increased oxidative stress. Increased POD activity in *Microcystis* LE-3, *Microcystis* SR, and *Anabaena* UTEX B377 specifically suggests that the intracellular levels of H_2_O_2_ in the strains were increased during exposure of MC-LR and ATX given this enzyme sequestrates and converts H_2_O_2_ to oxygen and water [48]. Similar responses have been observed in other phytoplankton during exposure to cyanotoxins [11,16,26].

SOD activity in all three cyanobacterial strains was increased in the presence of MC-LR, ATX, and MC-LR + ATX, signifying an increase in levels of the superoxide molecule, as SOD converts superoxide to H_2_O_2_, and subsequently, the H_2_O_2_ is transformed to water and oxygen by catalase and POD [50]. Given the increased activity of the enzyme, GST, in strains of *Microcystis*, it is seems likely involved in the detoxification of ATX and MC-LR. Several studies have shown that GST is actively involved in the detoxification and biotransformation of cyanotoxins into molecules that are less toxic to the cell [11,14,16,17,18,26,51]. The detoxification and biotransformation of cyanotoxins in cells is carried out via a conjugation process that is catalyzed by GST using the reduced form of glutathione [52,53]. Together, these findings support the hypothesis that cyanobacteria displayed increased enzymatic activity to protect against ROS during cyanotoxin exposure.

In contrast, to the three cyanobacterial strains that upregulated enzymes to combat oxidative stress when exposed to ATX and MC-LR, in *S. capricornutum* exhibited unchanged or lower POD, SOD and GST activities in the cyanotoxins treatments compared to unamended controls. This reduction of antioxidant enzyme activities in the green microalga is indicative of reduced ROS stress was consistent with the increased growth, chlorophyll-a content, and Fv/Fm observed in the present study when exposed to these cyanotoxins (Figure S3). Together, these imply the chlorophyte was in a better physiological state in the presence of the cyanotoxins [11,16,26,50] and dedicated cellular energy to growth rather than combating oxidative stress. These findings are also consistent with the hypothesis that *S. capricornutum* employed extracellular enzymes to degrade and use the cyanotoxins for nutrition [43,44,54], perhaps circumventing the need to upregulate antioxidant enzymes.

Microcystins have been shown to have multiple physiological functionalities in *Microcystis*, specifically aiding colony formation and potentially serving as an anti-oxidant [38,55,56]. Environmental conditions that reduce growth rates of *Microcystis* generally reduce microcystin production and consequently intracellular and extracellular concentrations [11,57,58,59,60]. In the present study, the only treatment with a significant effect on intracellular MCs content of *Microcystis* LE-3 was the MC-LR + ATX treatment, which resulted in higher production of intracellular microcystins. The increase in intracellular MCs content may have been an effort to increase the resistance/tolerance [38] of *Microcystis* LE-3 to the presence of exogenous cyanotoxins. Since it has been specifically hypothesized that microcystins may serve a critical role in defense against reactive oxygen species [55,56], the increase MC production may protect cells against external oxidative stress from the combined cyanotoxin treatment.

In the present study, ATX treatments (25 µg L^−1^ ATX, and 25 µg L^−1^ MC-LR combined with 25 µg L^−1^ ATX) significantly inhibited nitrogen fixation by *Anabaena* UTEX B377. Given that nitrogen fixation by diazotrophic cyanobacteria contributes significantly to the nitrogen supply of water bodies [61] it would appear that high levels of ATX can disrupt this important ecosystem function. Notably, 25 µg L^−1^ ATX elicited increasing activities of antioxidant enzymes such as POD, catalase (CAT), and GST while concurrently decreasing nitrogen fixation (Figure S4). The increase in activities of these enzymes as stated above is indicative of increased ROS production and oxidative stress, and both conditions negatively affect nitrogen fixation in cyanobacteria [62,63]. Increased ROS production alters the metabolic roles of nitrogenase by changing its structure and inhibiting the synthesis of the enzyme in diazotrophic cyanobacteria [62]. Furthermore, the expression of nitrogenase structural genes (*nifKDH*), and the biosynthesis of the *nifH* encoded Fe-protein are very susceptible to increased levels of cellular radicals [63]. Given the large amounts of energy needed to fix N, an investment in synthesizing enzymes to protect against ROS may inhibit an investment of cellular energy into diazotrophy. Once again, there may be a need to propose some sort of explanation for why *Anabaena* is not poisoning itself by producing anatoxin. The presence of ATX, although produced by diazotrophs such as *Anabaena*, *Aphanizomenon*, *Cylindrospermum*, and the benthic *Oscillatoria*, *Microcoleus*, and *Phormidium* [27], may partly control the form and availability of nitrogen in aquatic ecosystems by inhibiting the growth and nitrogen fixing capabilities diazotrophic cyanobacteria.

Collectively, this study has important ecological implications with regard to phytoplankton species succession. While dense blooms of toxic cyanobacteria can contain high levels of cyanotoxins that remain largely bound intracellularly [64], the sudden termination of such blooms by viral or bacterial lysis or programmed cell death [55] can often result in the sudden release of cyanotoxins into the dissolved pool [64]. Our introduction of high levels of cyanotoxins during our experiments simulated such an event and suggests that such a release might suppress the abundance of strains of *Microcystis* or *Anabaena*, but can stimulate the growth of the chlorophyte, *S. capricornutum*. Prior studies have demonstrated how allelochemicals and shifting nutrient regimes can favor succession between *Microcystis* or *Anabaena* [65]. This study demonstrates that the collapse of toxigenic cyanobacterial blooms with high levels of toxins could facilitate a transition toward eukaryotic algae such as *S. capricornutum*, a common successional pattern in freshwater ecosystems [2]. The concentration of the cyanotoxins used in the present study simulates those commonly found during cyanobacteria bloom events [28,32,66], which means lower levels of ATX and MC-LR will be detected most of the time in natural aquatic ecosystems. Therefore, the effect of high cyanotoxins concentrations on the structure and dynamics of phytoplankton community in environments with less frequent bloom events will probably be temporary and acute. 

## 4. Conclusions

Our results demonstrate that MC-LR and ATX can influence the growth and physiology of eukaryotic and prokaryotic phytoplankton. The ATX treatments significantly inhibited nitrogen fixation by *Anabaena* UTEX B377, which could have implications for the form and availability of nitrogen in aquatic ecosystems. Our results also show that ATX and MC-LR were stimulatory to *S. capricornutum* and thus may facilitate its dominance following the collapse of toxic cyanobacteria blooms. These results demonstrate that the presence of more than one cyanotoxin in the environment can have synergistic or antagonistic negative and positive effects on several physiological and successional processes depending on the toxin type or combination and strain/species of phytoplankton. Given that these outcomes could not have been predicted by the study of only one toxin in isolation, additional, future studies of the effects of multiple toxins produced by harmful algal and cyanobacterial blooms on aquatic life are clearly warranted.

## 5. Materials and Methods

The phytoplankton investigated in the present study included the cyanobacteria *Microcystis aeruginosa* strain LE-3 (*Microcystis* LE-3) isolated from Lake Erie, *Microcystis* sp. strain SR (*Microcystis* SR) isolated from the Sassafras River, MD, USA, and *Anabaena variabilis* (a.k.a. *Trichormus variabilis*) strain UTEX B377 (*Anabaena* UTEX B377) which is of unknown origin, and the chlorophyte *Selenastrum capricornutum* which is also of unknown origin. *Microcystis* SR and *Microcystis* LE-3 produce microcystin, while *Anabaena* UTEX B377 and *S. capricornutum* do not produce toxins. Experimental cultures were maintained in BG11 medium (pH 7.4) under controlled conditions (40 μmol m^−2^ s^−1^ Light intensity; 14:10 light:dark photoperiod; and 23 ± 1 °C temperature).

Microcystin-LR (≥98% purity) and ATX (>98% purity) were purchased from Cayman Chemical (Ann Arbor, MI, USA). Exponential phase cultures of the different strains (initial cell density of ~1 × 10^5^ cells mL^−1^) were spiked with 25 µg L^−1^ ATX, 25 µg L^−1^ MC-LR, and 25 µg L^−1^ MC-LR combined with 25 µg L^−1^ ATX (MC-LR + ATX) in 500 mL flasks having 250 mL of culture. The concentrations of ATX and MC-LR are within the range of concentrations detected in natural aquatic ecosystems during dense cyanobacterial blooms [28,32,66]. Microcystin-LR was selected because it is the most common microcystin congener reported worldwide [33]. Cultures were incubated for four days after toxin addition during which samples were collected to quantify cell density, maximum quantum efficiency of photosystem II, oxidative stress, and cyanotoxins on days 0, 2, and 4. All experiments were carried out in triplicate flasks for each treatment.

Cell densities of *Anabaena* UTEX B377 were measured with a gridded Sedgwick-Rafter counting chamber by enumerating at least 200 cells per sample. Cell counts of *Microcystis* SR, *Microcystis* LE-3, and *S. capricornutum* were carried out using a flow cytometer (CytoFLEX, Beckman Coulter, Brea, CA, USA) per the manufacturer’s instruction. Data acquisition and analysis were performed with the CytExpert Software (Beckman Coulter, Brea, CA, USA). Chlorophyll *a* concentration was quantified as per Parsons et al. [67] on a Turner Designs TD-700 fluorometer (Turner Designs, San Jose, CA, USA). Changes in maximum quantum efficiency of photosystem II were measured using the fluorescence enhanced by DCMU (3,4-dichlorophenyl-1,1-dimethylurea) (Fm) and in vivo fluorescence (F_i_) with an EM filter of >665 nm and EX filter of 340–500 nm on a Turner Designs TD-700 fluorometer [68,69]. The blank was corrected with BG11 medium during all fluorescence measurements. Total, dissolved, and particulate microcystin content was extracted using the Abraxis Quiklyse^TM^ cell lysis kit (Abraxis Inc., Warminster, PA, USA) for Microcystins/Nodularins and quantified using Abraxis Microcystin/Nodularian (ADDA) ELISA kit (Abraxis Inc., Warminster, PA, USA) per the manufacturer’s instructions, measured on SpectraMax Plus 384 plate-reading spectrophotometer (Molecular Devices, San Jose, CA, USA). Specifically, whole water was analyzed to quantify total microcystins, 10 mL samples were filtered via combusted glass fiber filters (GF/C; 2 h at 450 °C) to quantify dissolved toxins, and the difference between the whole and filtered samples representing particulate toxins. Filtered and whole water samples were lysed with an Abraxis Quiklyse^TM^ cell lysis kit, following the manufacturer’s instructions. Biomass derived from 40 mL of each phytoplankton culture under the different treatment conditions was extracted for antioxidant enzyme activity assays in phosphate buffer (0.1 M, pH 6.5, 1% *w*/*v* polyvinylpyrrolidone). The solution was vortexed to homogenize the biomass, sonicated, centrifuged at 10,000× *g* and 0 °C for 10 min, and the supernatant kept at −20 °C for biochemical assays. The activity of the anti-oxidant enzymes, superoxide dismutase (SOD), peroxidase (POD), and glutathione S-transferase (GST) was measured using CAT100, 19160, and CS0410 Sigma ELISA kits (Sigma-Aldrich, St. Louis, MO, USA), respectively, following the instructions of the manufacturer.

Nitrogen fixation by *Anabaena* UTEX B377, the only diazotrophic species in this study, was estimated using the acetylene reduction assay (ARA) outlined by Capone [70] with gas chromatography (GC), and calculated as the concentration of acetylene reduced to ethylene per hour (nmol cell^−1^ h^−1^) [71]. Specifically, 5 mL aliquots of *Anabaena* UTEX B377 cultures were sealed within 10 mL glass vials with a rubber septum to which acetylene was added to the gas phase and the vials were incubated for 4 h at 40 μmol m^−2^ s^−1^ and 23 ± 1 °C. After incubation, the ethylene content in the gas phase was measured using a Thermo Scientific Trace 1310 GC-FID system and the chromatographic ethylene peak area was calculated with the Chromeleon v7.2 Chromatography Data System (CDS) software (Thermo Scientific, Waltham, MA, USA). The amount of ethylene produced by *Anabaena* UTEX B377 was calculated from an ethylene standard curve and converted to N_2_ fixation rates using the assumption that each molecule of ethylene produced was assumed to be equivalent to 4 molecules of N_2_ fixed [72].

The data obtained was subjected to two-way analysis of variance (ANOVA) and significantly different means were separated using the Tukey’s HSD post hoc test at 0.05 significance level. Normality and homogeneity of the data were evaluated using the Shapiro-Wilk and Levene’s tests, respectively. The relationship between the different response parameters and the treatment conditions was evaluated using principal components analysis (PCA) with a correlation matrix. Prior to carrying out the PCA, measurements were converted to Z-scores whereby individual responses were standardized by subtracting them from the mean response of the parameters and subsequently dividing by their respective standard deviations. ANOVA and Tukey HSD tests were conducted in R statistical analysis software (v.3.5.0, The R Foundation for Statistical Computing, Welthandelsplatz, Vienna, Austria) and the PCA was created using PAST v.3.20 (Hammer & Harper, Problemveien, Oslo, Norway).

## Supplementary Materials

The following are available online at http://www.mdpi.com/2072-6651/11/1/43/s1, Figure S1: PCA biplot showing the relationship between the physiological responses of *Microcystis aeruginosa* LE-3 to different anatoxin-a (ATX) and microcystin-LR (MC-LR) treatments. SOD = Superoxide dismutase activity, POD = peroxidase activity, GST = Glutathione S-transferase activity, Chla-Cell = Chlorophyll content per cell, Chla-mL = Chlorophyll content per mL, Fv/Fm = maximum quantum efficiency of photosystem II. Figure S2: PCA biplot showing the relationship between the physiological responses of *Microcystis* sp. SR as a function of different anatoxin-a (ATX) and microcystin-LR (MC-LR) treatments. SOD = Superoxide dismutase activity, POD = peroxidase activity, GST = Glutathione S-transferase activity, Chla-Cell = Chlorophyll content per cell, Chla-mL = Chlorophyll content per mL, Fv/Fm = maximum quantum efficiency of photosystem II. Figure S3: PCA biplot showing the relationship between the physiological responses of *Selenastrum capricornutum* to different anatoxin-a (ATX) and microcystin-LR (MC-LR) treatments. SOD = Superoxide dismutase activity, POD = peroxidase activity, GST = Glutathione S-transferase activity, Chla-Cell = Chlorophyll content per cell, Chla-mL = Chlorophyll content per mL, Fv/Fm = maximum quantum efficiency of photosystem II. Figure S4: PCA biplot showing the relationship between the physiological responses of *Anabaena variabilis* UTEX B377 during exposure to different anatoxin-a (ATX) and microcystin-LR (MC-LR) treatments. SOD = Superoxide dismutase activity, POD = peroxidase activity, GST = Glutathione S-transferase activity, Chla-Cell = Chlorophyll content per cell, Chla-mL = Chlorophyll content per mL, Fv/Fm = maximum quantum efficiency of photosystem II.
