# Supplementary material for: The Individual and Combined Effects of the Cyanotoxins, Anatoxin-a and Microcystin-LR, on the Growth, Toxin Production, and Nitrogen Fixation of Prokaryotic and Eukaryotic Algae
Source: Toxins (Basel). 2019 Jan 15;11(1):43. doi: 10.3390/toxins11010043 (PMC6357180; doi:10.3390/toxins11010043)
Supplement: Supplementary file 1 [file toxins-11-00043-s001.pdf]

Supplementary Materials: The Individual and Combined Effects of the Cyanotoxins, Anatoxin-a and Microcystin-LR, on the Growth, Toxin Production, and Nitrogen Fixation of Prokaryotic and Eukaryotic Algae

Mathias Ahii Chia, Benjamin J. Kramer, Jennifer G. Jankowiak, Maria do Carmo Bittencourt-Oliveira and Christopher J. Gobler

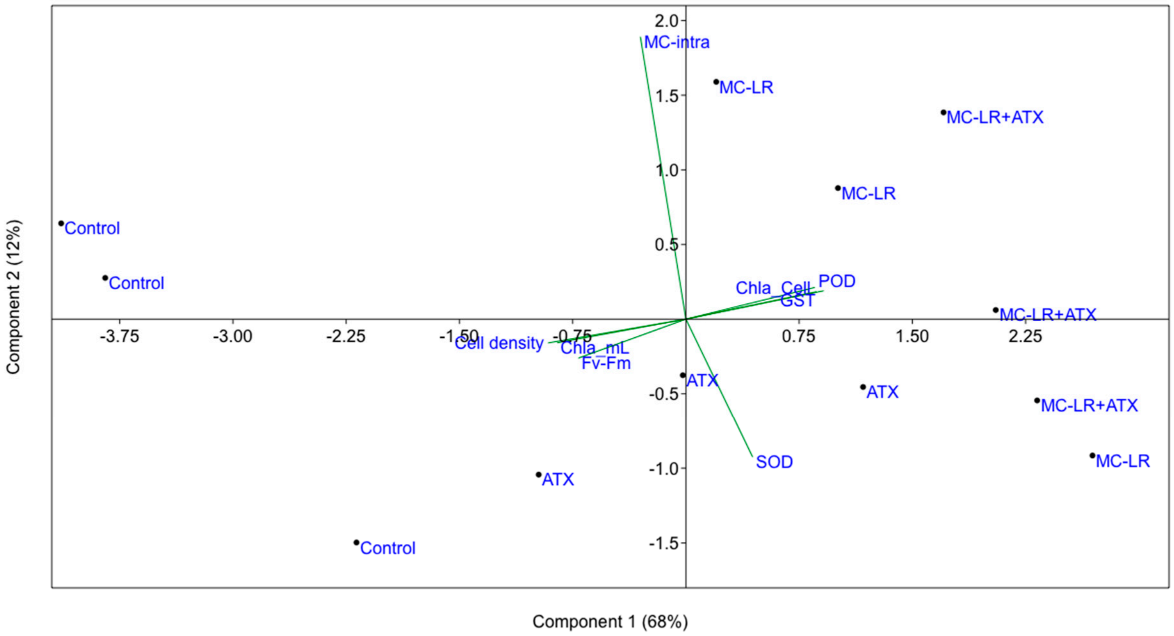

Figure S1. PCA biplot showing the relationship between the physiological responses of *Microcystis aeruginosa* LE-3 to different anatoxin-a (ATX) and microcystin-LR (MC-LR) treatments. SOD = Superoxide dismutase activity, POD = peroxidase activity, GST = Glutathione S-transferase activity, Chla-Cell = Chlorophyll content per cell, Chla-mL = Chlorophyll content per mL, Fv/Fm = maximum quantum efficiency of photosystem II.

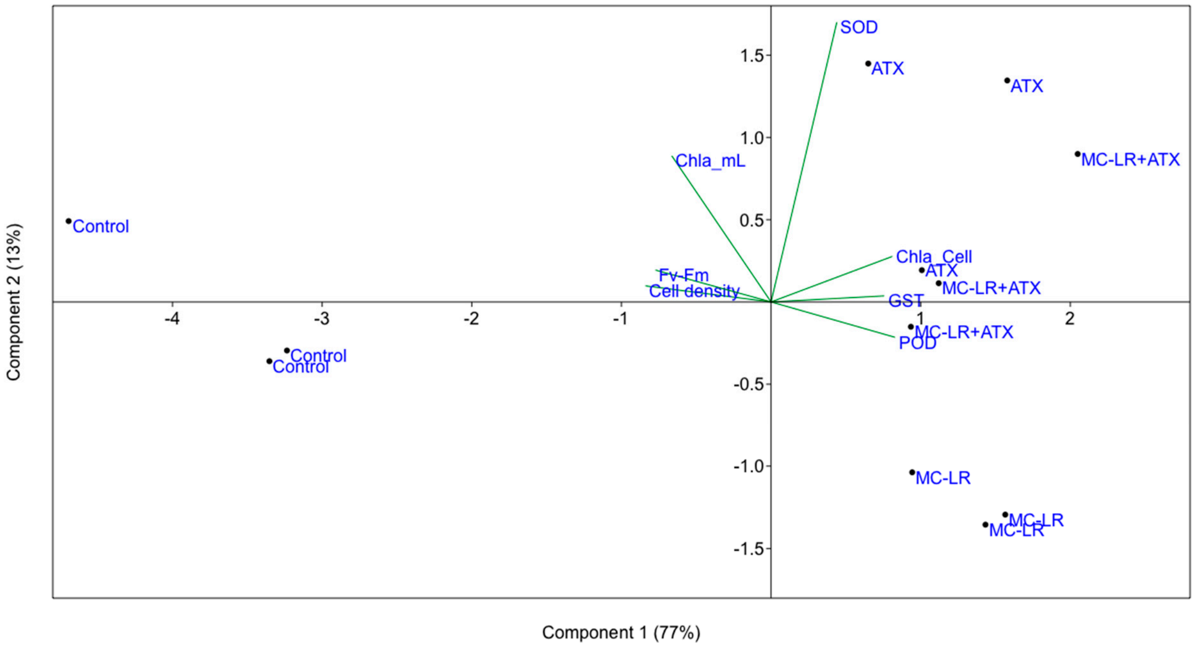

Figure S2. PCA biplot showing the relationship between the physiological responses of *Microcystis* sp. SR as a function of different anatoxin-a (ATX) and microcystin-LR (MC-LR) treatments. SOD = Superoxide dismutase activity, POD = peroxidase activity, GST = Glutathione S-transferase activity, Chla-Cell = Chlorophyll content per cell, Chla-mL = Chlorophyll content per mL, Fv/Fm = maximum quantum efficiency of photosystem II.

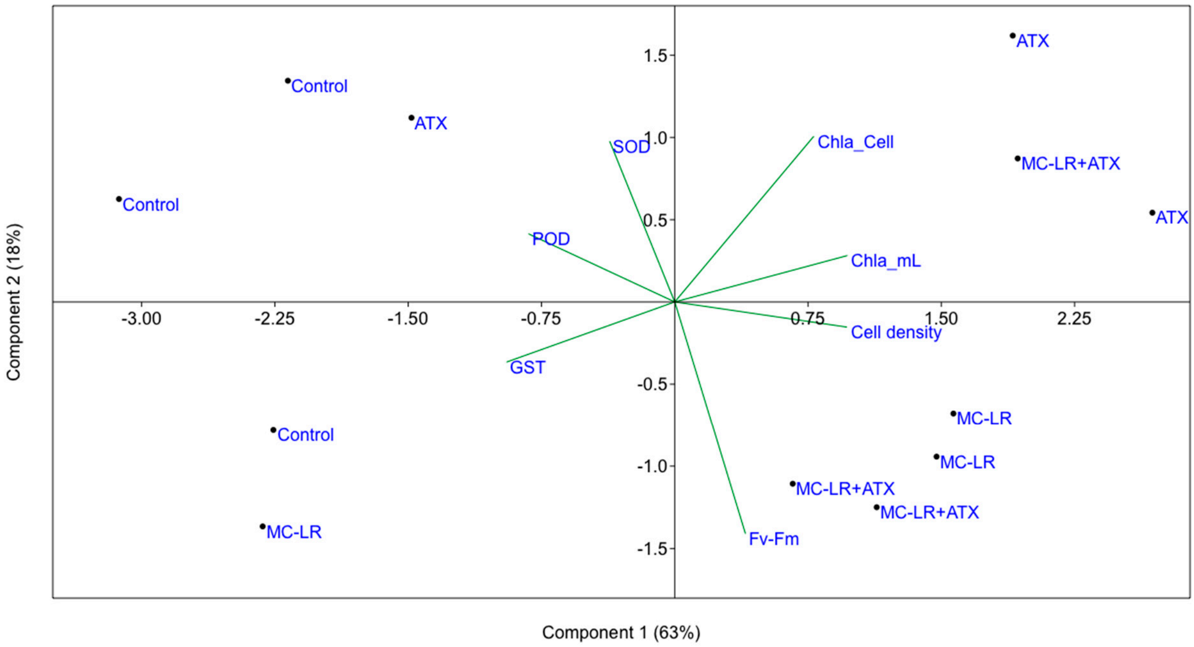

Figure S3. PCA biplot showing the relationship between the physiological responses of *Selenastrum capricornutum* to different anatoxin-a (ATX) and microcystin-LR (MC-LR) treatments. SOD = Superoxide dismutase activity, POD = peroxidase activity, GST = Glutathione S-transferase activity, Chla-Cell = Chlorophyll content per cell, Chla-mL = Chlorophyll content per mL, Fv/Fm = maximum quantum efficiency of photosystem II.

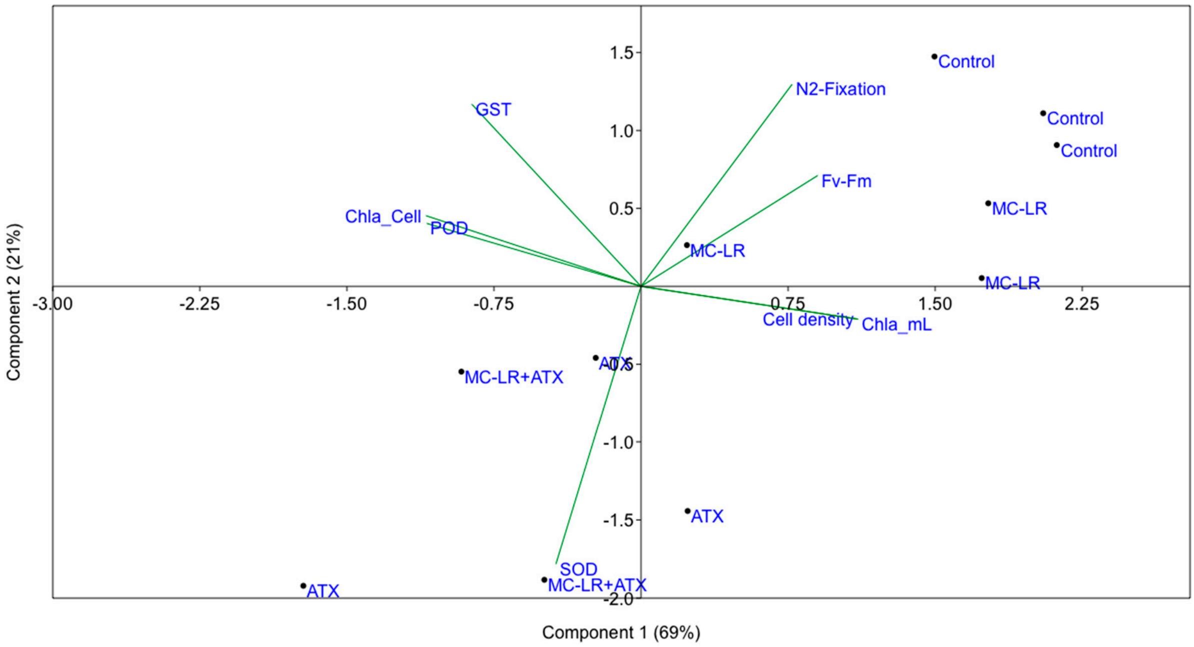

Figure S4. PCA biplot showing the relationship between the physiological responses of *Anabaena variabilis* UTEX B377 during exposure to different anatoxin-a (ATX) and microcystin-LR (MC-LR) treatments. SOD = Superoxide dismutase activity, POD = peroxidase activity, GST = Glutathione S-transferase activity, Chla-Cell = Chlorophyll content per cell, Chla-mL = Chlorophyll content per mL, Fv/Fm = maximum quantum efficiency of photosystem II.
